# A Novel CNN–ViT Model with Cascade Upsampling for Efficient Crack Segmentation

**DOI:** 10.3390/s26051667

**Published:** 2026-03-06

**Authors:** Ahmed Tibermacine, Imad Eddine Tibermacine, Zineddine S. Kahhoul, Ilyes Naidji, Abdelaziz Rabehi, Mustapha Habib

**Affiliations:** 1 LESIA Laboratory, Department of Computer Science, University of Biskra, Biskra 07000, Algeria; 2 Department of Computer, Automation and Management Engineering, Sapienza University of Rome, 00185 Rome, Italy; 3 VSC Laboratory, University of Biskra, Biskra 07000, Algeria; zineddine.kahhoul@univ-biskra.dz; 4 RLP Laboratory, Department of Computer Science, University of Biskra, Biskra 07000, Algeria; 5 Laboratory of Telecommunications and Smart Systems, Faculty of Sciences and Technologies, University of Djelfa, Djelfa 17000, Algeria; 6 Division of Building Technology and Design, Department of Civil and Architectural Engineering, KTH Royal Institute of Technology, 11428 Stockholm, Sweden

**Keywords:** crack segmentation, vision transformer, convolutional neural networks, structural health monitoring, deep learning, edge computing, optical digital imaging

## Abstract

Accurate crack segmentation in civil infrastructure imagery remains challenging because of the prevalence of thin, low-contrast, and spatially discontinuous defects that often appear amid textured surfaces, shadows, and acquisition noise. Although Transformer-based models improve global context modeling, many existing solutions incur substantial computational and memory overhead, which limits their use in practical, resource-constrained inspection settings. In this work, we introduce an efficient hybrid segmentation architecture that combines a convolutional encoder for high-fidelity local representation with a lightweight Transformer bottleneck for global dependency modeling, followed by a progressive decoder that restores spatial resolution through multi-level skip-feature fusion. To better accommodate severe foreground sparsity and preserve fine crack structures, the framework is trained with a composite Dice–Binary Cross-Entropy objective and employs a tokenization strategy designed to preserve fine spatial details while enabling efficient global context modeling. We validate the proposed approach on four public benchmarks, demonstrating consistent improvements over representative convolutional, Transformer-based, and hybrid baselines, while ablation studies confirm the contribution of each design component. Finally, runtime profiling shows favorable latency and memory characteristics, supporting real-time or near real-time deployment on embedded and edge inspection platforms.

## 1. Introduction

Maintaining structural integrity in civil infrastructure is critical for public safety, economic stability, and societal sustainability. Structures face environmental weathering, material degradation, and operational stress throughout their service life, leading to surface cracks that often signal deeper deficiencies. If not identified and repaired promptly, these defects can propagate and cause catastrophic failure. Traditional manual inspection methods are labor-intensive, subjective, and inefficient, especially for large-scale or hazardous environments. This drives demand for automated crack detection systems that are rigorous, scalable, and reliable under real-world conditions.

Pixel-level segmentation is essential compared to detection-based approaches because it provides the morphological information needed for structural health assessment. While detection can locate cracks, critical indicators of structural failure, including width, length, orientation, and connectivity, can only be measured from precise segmentation masks. Width progression over time requires exact pixel-level boundaries, and understanding whether cracks are connected or isolated informs repair decisions. Segmentation also enables quantitative analysis that supports automated condition rating systems. Thus, while detection suffices for simple screening, segmentation is necessary for comprehensive structural evaluation in real-world infrastructure monitoring.

In recent years, deep learning has emerged as a transformative force in the field of visual crack detection, largely driven by the widespread adoption of CNNs for image segmentation tasks [1]. Contemporary CNNs-based segmentation frameworks have shown robust capabilities in extracting granular features and accurately delineating structural boundaries, even when confronted with challenging scenarios characterized by high noise levels or poor image contrast [2]. Despite these successes, CNNs are fundamentally constrained by the locality of their receptive fields, a characteristic that inherently limits their ability to model long-range dependencies effectively [3]. This limitation becomes particularly acute when the objective is the detection of thin, spatially fragmented, or widely dispersed crack patterns [4]. Furthermore, standard CNN architectures often exhibit performance degradation in scenarios involving variable lighting conditions, complex background textures, or surface noise—phenomena that are ubiquitous in practical infrastructure inspection [5].

To mitigate these inherent limitations, Vision Transformers (ViTs) have recently risen to prominence, distinguished by their ability to model global relationships via self-attention mechanisms. Transformer-based architectures [6,7] have achieved state-of-the-art performance across a spectrum of image segmentation applications. Within the specific domain of crack detection, specialized models [8,9] have empirically demonstrated the advantages of global contextual reasoning in capturing complex crack morphologies. Despite these advancements, a significant number of existing Transformer-based approaches suffer from a reduction in spatial granularity, a consequence of coarse patch tokenization. Moreover, their substantial computational complexity presents a formidable barrier to real-time deployment on edge devices and embedded platforms.

Although prior research has endeavored to address isolated challenges—such as class imbalance, morphological variability, and the precise localization of micro-defects—there remains a paucity of approaches that offer a truly comprehensive solution. Specifically, there is a notable absence of models that simultaneously optimize segmentation accuracy, computational efficiency, and deployment feasibility. While hybrid CNN–Transformer architectures have been proposed, many compromise performance in favor of simplicity or introduce excessive complexity without adequately addressing task-specific requirements and the constraints of practical deployment.

In this study, we propose a lightweight hybrid architecture that synergistically integrates CNN and Transformer components to deliver accurate and efficient crack segmentation under realistic operational conditions. Our model is constructed upon an encoder–decoder framework, featuring a convolutional encoder tasked with capturing fine-grained local features and a lightweight Transformer bottleneck designed to model global context. The decoder employs a cascade upsampling strategy, supported by refined skip connections that are essential for preserving spatial continuity and enhancing boundary accuracy. In contrast to standard hybrid models [7,10], our architecture introduces a series of task-specific adaptations. These include optimized patch sizing derived from rigorous receptive field analysis, transformer tuning to ensure structural consistency, and a decoder specifically engineered to handle thin and fragmented crack structures with high fidelity.

We have subjected the proposed model to extensive evaluation using four publicly available benchmark datasets [11,12,13,14]. These datasets collectively encompass a diverse range of environments, surface typologies, and crack geometries, thereby providing a robust test bed for assessing generalization capabilities. Our model consistently outperforms established baselines [6,8,15] across key metrics, including the Dice coefficient, Intersection-over-Union (IoU), and pixel accuracy. Furthermore, we evaluate the real-time performance and resource efficiency of the model on constrained hardware platforms. The results demonstrate the model’s suitability for embedded applications, showcasing a compact architecture characterized by a low memory footprint, reduced computational complexity, and the capability for real-time inference on resource-constrained platforms.

The paper is structured as follows: Section 2 provides a comprehensive review of related research in the field of crack segmentation. Section 3 elucidates the details of the proposed architecture. Section 4 delineates the experimental evaluations and methodologies. Section 5 offers an in-depth discussion regarding ablation studies, benchmarking comparisons, and deployment considerations. Finally, Section 6 concludes the paper and outlines potential avenues for future research.

## 2. Related Work

The field of automatic crack segmentation within civil infrastructure has undergone a significant evolution, transitioning from classical image processing techniques to sophisticated deep learning-based frameworks. Traditional methodologies predominantly relied upon handcrafted features, employing techniques such as edge detectors, morphological filtering, and thresholding [16,17]. While these techniques offered computational efficiency, they were plagued by a high sensitivity to noise, lighting variations, and diverse surface textures, factors which severely limited their robustness in real-world applications.

The advent of CNNs marked a paradigm shift in the domain of crack detection [4,18]. Early CNN-based approaches functioned primarily on local image patches [1], a strategy that constrained the model’s contextual understanding. The introduction of Fully Convolutional Networks (FCNs) [19] and encoder–decoder architectures, most notably U-Net [3], facilitated dense pixel-wise prediction. This advancement significantly improved the ability to segment thin and fragmented cracks [20,21]. The skip connections integral to these models assist in recovering spatial details lost during the downsampling process. However, CNNs remain fundamentally limited by their local receptive fields, which reduces their capacity to capture the long-range dependencies essential for detecting discontinuous or branching crack patterns. Multiscale architectures such as DeepCrack [22] and hierarchical frameworks like SC-CrackSeg [23] have attempted to address this by combining features at varying levels of abstraction, yet their performance is often compromised in cluttered environments.

Vision Transformers [24] have introduced a powerful alternative by modeling global interactions through self-attention mechanisms. Transformer-based models, including SETR [25], Swin Transformer [26], and SegFormer [6], have achieved state-of-the-art performance in general semantic segmentation tasks. Within the specific domain of crack detection, specialized architectures such as CrackFormer [8], CrackSegFormer [9], and APTNet utilize ViTs to detect complex, low-contrast, and irregular crack structures. Although these models exhibit strong contextual reasoning, they frequently suffer from excessive memory usage, slower inference speeds, and spatial resolution degradation due to patch embedding, rendering them suboptimal for real-time and edge computing scenarios.

To overcome the respective limitations of pure CNN or Transformer models, hybrid architectures have emerged. These systems combine the local feature extraction strengths of CNNs with the global modeling capabilities of Transformers. TransUNet [10], Swin-UNet [7], and MedT [27] exemplify this hybrid trend. Originally designed for medical imaging, these models have shown promise in infrastructure inspection contexts [28,29]. However, many remain computationally intensive, limiting their applicability in field deployments where hardware resources and energy supplies are constrained.

In response to these challenges, a growing body of work has emphasized efficiency and lightweight deployment. Zhang et al. proposed an edge-optimized model for on-device crack segmentation [30] and surveyed quantization techniques aimed at reducing inference latency and memory usage [5]. Their work on UAV-based inspection using TinyML frameworks [31] demonstrates the real-world potential of compact, embedded crack detection solutions. However, these lightweight models often require a trade-off between accuracy and efficiency.

More recently, state-space models such as Mamba [32] have been introduced to tackle the challenge of modeling long-range dependencies with linear time and space complexity. Unlike traditional attention mechanisms, Mamba-based architectures operate through learned recurrence and convolutional filtering, enabling scalable sequence processing. Swin-Mamba [33] and Mamba-Seg [34] have demonstrated success in high-throughput segmentation tasks. In the context of crack segmentation, Zuo et al. [35] developed a topology-aware Mamba model that incorporates structural priors to improve the continuity of detected cracks, while Chen et al. [36] proposed a lightweight Mamba variant optimized for UAV and edge deployment. Furthermore, CrackMamba [37] integrates a Frangi-filter normalization strategy with Mamba to improve the detection of narrow and faint cracks.

Although Mamba-based models offer efficient long-range dependency modeling, they often compromise spatial detail due to sequential processing and limited feature expressiveness compared to CNNs. Their architectures typically lack multiscale fusion and skip connections, which are essential for segmenting fine, discontinuous cracks in complex backgrounds [38,39]. Moreover, most are not validated across diverse datasets or optimized for heterogeneous edge deployments [39]. To address these gaps, we propose a hybrid CNN–Transformer model that combines local texture extraction, lightweight global context modeling, and a cascade decoder with skip connections for precise boundary recovery. This architecture achieves a favorable balance between accuracy, efficiency, and deployability, demonstrating superior generalization and real-time crack segmentation performance under diverse infrastructure conditions compared to recent state-space sequence modeling approaches.

## 3. Proposed Method

**Figure 1:**
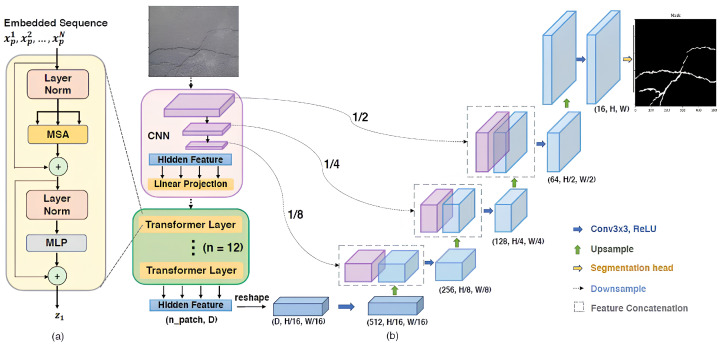
Overview of the proposed hybrid CNN–Transformer architecture. (**a**) Encoder-decoder pathway. (**b**) Transformer bottleneck details.

This section introduces a lightweight hybrid architecture tailored for crack segmentation, combining a CNN-based encoder, a Transformer bottleneck for global context, and a streamlined decoder with skip connections for accurate and efficient spatial reconstruction. The full model design is illustrated in Figure 1.

### 3.1. CNN-Based Encoder: Local Feature Extraction

The encoder is responsible for learning rich local representations of the input image by progressively extracting spatially localized features. It is implemented as a deep convolutional stack composed of four stages, where each stage includes convolutional operations followed by non-linear activations and spatial downsampling. This hierarchical structure enables the network to capture increasingly abstract patterns, ranging from low-level edges to mid-level textures, that are critical for representing the fine, irregular structures characteristic of cracks.

Each convolutional layer performs a discrete convolution operation defined as:

(1) $$F_{i,j}^{\left(l\right)}=\sigma \left(\sum_{m=1}^{C_{l-1}}\sum_{p=1}^{k}\sum_{q=1}^{k}W_{m,p,q}^{\left(l\right)}\cdot X_{i+p,j+q}^{\left(l-1\right)}+b^{\left(l\right)}\right)$$

where:

- $X^{\left(l-1\right)}$ is the input feature map at layer (l−1),
- $W^{\left(l\right)}$ are the learnable convolutional kernel weights of size k×k,
- $C_{l-1}$ is the number of input channels at layer (l−1),
- σ(·) is the ReLU activation function,
- $F_{i,j}^{\left(l\right)}$ is the output activation at spatial location (i,j).

In our design, each convolutional block employs a 3×3 kernel, which provides an optimal trade-off between spatial detail and computational efficiency. To control spatial dimensionality and expand the receptive field, we apply 2×2 max-pooling after each block:

(2) $$P_{i,j}=\max_{(u,v)\in \Omega }F_{2i+u,2j+v}$$

where Ω is the pooling window of size 2×2.

The encoder progressively reduces the input resolution from 512×512 to 32×32 while increasing the number of feature channels from 64 to 512, producing compact yet expressive feature representations. Specifically, the encoder produces feature maps at four resolutions: 256×256 with 64 channels (stage 1), 128×128 with 128 channels (stage 2), 64×64 with 256 channels (stage 3), and 32×32 with 512 channels (stage 4). Importantly, we selected a depth of four downsampling stages to strike a balance between two competing objectives: (1) preserving sufficient spatial resolution to detect narrow cracks, which are often less than 5 pixels wide, and (2) ensuring a receptive field that is large enough to capture the contextual patterns necessary for modeling crack continuity and morphology.

Deeper encoders (e.g., five or more stages) were experimentally found to degrade crack localization performance due to excessive spatial reduction, while shallower encoders (e.g., three stages) failed to capture sufficient abstraction. The chosen configuration, therefore, reflects a morphology-aware design choice optimized for the typical visual scale of cracks. The resulting multi-scale feature maps encode detailed spatial cues, with stage 2 features at 128×128 resolution serving as input to the Transformer bottleneck, that are passed to the decoder via skip connections to augment them with global contextual reasoning.

### 3.2. Transformer Bottleneck: Global Context Modeling

While CNNs effectively capture local patterns, they are inherently limited by their localized receptive fields, making it difficult to model long-range dependencies or contextual relationships across disconnected crack fragments. To address this, we employ a Vision Transformer bottleneck that operates on tokenized image patches and models full-image dependencies via self-attention mechanisms.

In our implementation, the transformer bottleneck operates on feature maps from an intermediate encoder stage rather than the final encoder output. Specifically, we extract feature maps after the second encoder stage at a resolution of 128×128 with 256 channels. This design choice preserves sufficient spatial detail for subsequent global context modeling while maintaining computational efficiency.

The input feature map of size H×W×C (where H=W=128 and C=256) is divided into *N* non-overlapping patches of size P×P, with P=16. The number of tokens is therefore:

(3) $$N=\frac{H\cdot W}{P^{2}}=\frac{128\times 128}{16\times 16}=64,$$

arranged in an 8×8 spatial grid. Each patch is flattened and linearly projected into a *D*-dimensional embedding space, where D=128, forming a sequence of tokens $X\in R^{N\times D}$. To retain spatial information, learnable positional encodings $E_{\mathrm{pos}}$ are added:

(4) $$Z_{0}=X+E_{\mathrm{pos}}$$

The token sequence $\left\{Z_{0}\right\}$ is then passed through *L* transformer layers. We employ L=4 transformer layers, each utilizing h=4 attention heads. The Multi-Head Self-Attention mechanism operates with a key dimension $d_{k}=D/h=32$ per head. Each layer consists of a Multi-Head Self-Attention (MSA) mechanism followed by a Feed-Forward Network (FFN) with an expansion factor of 4 (i.e., FFN hidden dimension = 512), incorporating residual connections and layer normalization (LN):

(5) $$Z^{\prime }=\mathrm{MSA}\left(\mathrm{LN}\left(Z_{l-1}\right)\right)+Z_{l-1}\;\mathrm{and}\;Z_{l}=\mathrm{FFN}\left(\mathrm{LN}\left(Z^{\prime }\right)\right)+Z^{\prime }$$

In the MSA module, each token attends to all others using the scaled dot-product attention:

(6) $$\mathrm{Attention}\left(Q,K,V\right)=\mathrm{softmax}\left(\frac{QK^{⊤}}{\sqrt{d_{k}}}\right)V$$

where $Q,K,V\in R^{N\times d_{k}}$ are learned linear projections of the input Z, and $d_{k}$ is the dimension of the key vectors.

This formulation allows the model to learn global relationships between spatially distant patches, which is essential for detecting discontinuous cracks that extend across the entire field of view. After *L* transformer layers, the sequence is reshaped back into a 2D feature map of size 8×8×128 and then upsampled and projected to match the dimensions required by the decoder stages. This reshaped feature map serves as the global context-enriched representation that is passed to the decoder for spatial reconstruction.

### 3.3. Cascade Decoder with Skip Connections

The decoder is responsible for reconstructing a high-resolution segmentation mask from the lower-resolution, globally contextualized representations output by the transformer bottleneck. It follows a cascade upsampling path consisting of bilinear upsampling layers, each followed by 3×3 convolutional operations and ReLU activations. This progressive upsampling allows the model to recover spatial resolution step by step, moving from coarse semantic maps to fine-grained segmentation.

Formally, let $Z_{l}$ be the decoder feature map at level *l*. Upsampling is applied as:

(7) $$Z_{l+1}^{↑}=\mathrm{Upsample}\left(Z_{l}\right),\;\mathrm{where}\;\mathrm{Upsample}\left(\cdot \right)\in R^{2H\times 2W\times C}$$

To enhance the decoder’s capacity to recover fine structures, skip connections are introduced from the encoder to corresponding decoder layers. At each level, the upsampled decoder feature map is concatenated with the encoder output at the same spatial resolution:

(8) $$F_{l}={\mathrm{Conv}}_{3\times 3}\left(\mathrm{Concat}(Z_{l+1}^{↑},E_{l})\right)$$

where $E_{l}$ is the encoder feature map at level *l*. This fusion provides both high-level semantic features and low-level spatial details, enabling accurate localization of thin, low-contrast cracks.

The final segmentation map is produced by applying a 1×1 convolution to reduce the number of channels to 1 (binary class), followed by a sigmoid activation to obtain per-pixel probabilities:

(9) $$\hat{Y}\left(x,y\right)=\sigma \left({\mathrm{Conv}}_{1\times 1}\left(F_{\mathrm{final}}\left(x,y\right)\right)\right)$$

where $\hat{Y}\left(x,y\right)\in \left[0,1\right]$ indicates the predicted probability of pixel (x,y) being part of a crack.

This cascade architecture, enriched by skip connections and upsampling, allows the decoder to produce high-resolution segmentation maps that are both spatially accurate and semantically coherent, which is critical in infrastructure monitoring tasks.

### 3.4. Architectural Innovations

The proposed model introduces several architectural innovations that are specifically designed to address the challenges of crack segmentation, such as thin structure preservation, spatial discontinuity, severe class imbalance, and deployment efficiency. These innovations span from architectural fusion strategies to efficient transformer configurations, collectively enabling robust segmentation under real-world conditions.

#### 3.4.1. Global–Local Feature Fusion

One of the key challenges in semantic segmentation is the need to integrate local spatial information with long-range contextual cues. CNNs are effective in capturing localized features due to their spatial inductive bias and translation invariance, while Transformers are adept at modeling global dependencies through self-attention mechanisms. However, each paradigm alone is insufficient: CNNs struggle with discontinuities, and Transformers may sacrifice fine-grained spatial resolution.

To overcome this, the proposed architecture adopts a dual-branch structure in which the encoder leverages convolutional blocks to learn local patterns $F_{\mathrm{CNN}}\in R^{H\times W\times C}$, and the bottleneck transformer module learns global dependencies among spatial patches $F_{\mathrm{ViT}}\in R^{N\times D}$ via token-wise attention. These features are integrated in the decoder through hierarchical skip connections and fusion blocks:

(10) $$F_{\mathrm{fusion}}^{\left(l\right)}={\mathrm{Conv}}_{3\times 3}\left(\mathrm{Concat}\left(F_{\mathrm{CNN}}^{\left(l\right)},F_{\mathrm{ViT}}^{\left(l\right)}\right)\right)$$

This design preserves low-level details necessary for delineating thin cracks, while simultaneously incorporating high-level structural context to capture disconnected or non-linear crack trajectories.

#### 3.4.2. Optimized Skip Connections

In pure Transformer architectures, the early tokenization and lack of inductive bias often lead to poor reconstruction of fine structures. To mitigate this, our model introduces multi-level skip connections between the encoder and decoder at all four spatial resolutions. At each level *l*, the upsampled decoder feature map $Z^{\left(l\right)}$ is fused with the encoder feature map $E^{\left(l\right)}$ via concatenation followed by convolution:

(11) $$U^{\left(l\right)}={\mathrm{Conv}}_{3\times 3}\left(\mathrm{Concat}\left(\mathrm{Upsample}\left(Z^{\left(l+1\right)}\right),E^{\left(l\right)}\right)\right)$$

This strategy allows precise spatial cues from the encoder to be reinjected into the decoding process, ensuring that fine crack boundaries are preserved during upsampling. Such skip-based fusion not only accelerates convergence but also improves gradient flow during backpropagation, especially in deeper networks.

#### 3.4.3. Lightweight ViT for Edge Deployment

To enable real-time use on resource-constrained devices, the model uses a lightweight Transformer that balances global context modeling with computational efficiency, avoiding large architectures while preserving long-range dependency modeling.

The transformer bottleneck operates on encoder stage-2 feature maps of size 128×128. These maps are partitioned into 16×16 patches, producing N=64 tokens arranged in an 8×8 grid. Each token is embedded into a D=128 dimensional space and processed by L=4 transformer layers with h=4 attention heads.

The computational complexity of the self-attention mechanism in our design is $O(N^{2}D)$ for standard full self-attention. However, with N=64 and D=128, the practical computational cost remains modest ($64^{2}\times 128\approx 524K$ operations per layer). It is important to clarify that we do not employ linear attention mechanisms; rather, our efficiency stems from the intentional design choice to tokenize at an intermediate resolution (128 × 128) with moderate embedding dimensions, keeping both *N* and *D* small while maintaining sufficient spatial granularity for effective global context modeling.

The lightweight Transformer layers are sufficient to capture essential global context while allowing the model to run at over 30 FPS on mid-range GPUs, enabling field deployment without the need for server-grade hardware.

#### 3.4.4. Crack-Aware Design

Crack segmentation presents a unique challenge due to the extremely narrow, discontinuous, and morphologically irregular nature of cracks, which often span just a few pixels in width and exhibit high shape variability. To accommodate this, our architecture is carefully tuned to respect both the spatial and contextual demands of crack morphology.

A critical design decision is determining where and how to apply the Transformer bottleneck. Rather than operating on the final encoder output (which would excessively reduce spatial resolution), we apply the Transformer to feature maps from encoder stage 2 at a resolution of 128×128. This intermediate representation preserves sufficient spatial detail while still providing a compact representation for global context modeling.

We select a patch size of P=16×16 for tokenizing these 128×128 feature maps, which offers an effective trade-off between attention span and spatial granularity. This configuration yields N=64 tokens arranged in an 8×8 spatial grid, providing sufficient positional resolution to model relationships across the image while maintaining computational efficiency. Larger patches (e.g., 32×32, which would yield only 16 tokens) were found to reduce sensitivity to fine crack structures, as they dilute local cues within a broader context window. Conversely, smaller patches (e.g., 8×8, yielding 256 tokens) increase spatial resolution but incur significant computational cost with diminishing gains in segmentation accuracy.

Let *r* denote the average crack width in pixels, where r<5 in typical concrete and asphalt defect imagery. We define an effective patch scale *P* satisfying:

(12) $$r<P<\frac{H_{\mathrm{token}}}{2}$$

where $H_{\mathrm{token}}$ is the spatial dimension of the feature map being tokenized.

In our design, $H_{\mathrm{token}}=128$ and P=16, satisfying 5<16<64. This condition ensures that each patch encompasses multiple crack width pixels (providing sufficient contextual information) while maintaining enough patches (8×8 grid) to model spatial relationships across the image. The chosen tokenization resolution and patch size, when combined with a 4-stage CNN encoder, guarantees a receptive field large enough to capture long-range crack continuity, while preserving fine-grained spatial detail through the skip connections that bypass the Transformer bottleneck.

Additionally, the decoder architecture includes multi-stage upsampling and skip connections from early encoder layers to recover fine-grained boundary information. Critically, these skip connections directly connect encoder stages 1 through 4 to corresponding decoder stages, bypassing the Transformer bottleneck entirely. This allows high-resolution spatial features to flow directly to the decoder, ensuring that thin crack structures are not lost during the tokenization and global context modeling process. These skip paths inject high-resolution spatial features that are essential for delineating thin and branching crack regions. The final prediction head employs a 1×1 convolution followed by a sigmoid activation to generate a dense, pixel-level crack probability map $\hat{Y}\left(x,y\right)\in \left[0,1\right]$.

This crack-aware design, which includes tokenization resolution, patch size selection, encoder depth, receptive field planning, and high-resolution fusion via skip connections, ensures that the model is well aligned with the morphological characteristics of real-world cracks. It is particularly effective in detecting micro-fractures, hairline cracks, and irregular patterns that are critical for structural health monitoring in practical scenarios.

## 4. Experimental Results

This section presents the experimental framework used to evaluate the proposed model, including dataset descriptions, training configurations, evaluation metrics, and detailed performance results across multiple benchmark datasets.

### 4.1. Datasets

We evaluate the proposed model on four diverse public datasets: Crack Segmentation Dataset [11], Crack500 [13], SDNET2018 [14], and DeepCrack [12]. These datasets were selected to comprehensively assess the model’s robustness and generalization across a wide range of imaging conditions, structural materials, and crack morphologies typically encountered in real-world civil infrastructure.

#### 4.1.1. Crack Segmentation Dataset

The Crack Segmentation Dataset [11] is a composite dataset merging approximately 11,200 images from 12 publicly available crack segmentation datasets. The dataset includes both crack-containing images and crack-free images. For our experiments, we used a subset of 2581 images containing cracks across concrete structures, walls, pavements, and bridge decks, each with a corresponding binary mask. This subset exhibits significant variability in illumination, occlusion, and material texture, with crack pixels representing approximately 4.7% of the total.

#### 4.1.2. Crack500

Crack500 [13] is a road-oriented benchmark dataset comprising 500 RGB images with corresponding binary segmentation masks. Captured under diverse real-world conditions, it features complex pavement textures, shadow occlusions, and challenging lighting variations, making it particularly suitable for evaluating model robustness to environmental noise and surface inconsistencies. All 500 images contain cracks; no crack-free images are included. Therefore, false positives are assessed on background regions within crack-containing images rather than on entirely negative samples.

#### 4.1.3. SDNET2018

The SDNET2018 dataset [14] contains 56,092 annotated images of concrete bridge decks, walls, and pavements, with each image resized to 256×256 pixels. Images feature cracks as narrow as 0.06 mm and various obstructions including shadows, surface roughness, and background debris. The dataset includes 8484 cracked and 47,608 non-cracked images [14], enabling false positive evaluation on entirely crack-free scenes—critical for real-world deployment where both cracked and intact surfaces appear.

#### 4.1.4. DeepCrack

The DeepCrack dataset [12] comprises 537 RGB images of pavements and walls with pixel-level crack annotations. Images feature challenging conditions including low contrast, poor continuity, and cluttered backgrounds. All images contain cracks; no crack-free (negative) samples are included. False positives are therefore evaluated on background regions within crack-containing images rather than on entirely non-cracked scenes, which should be considered when interpreting deployment performance.

#### 4.1.5. Preprocessing and Augmentation

To ensure consistent training and evaluation across datasets, all images were resized to 512×512 using bilinear interpolation. Segmentation masks were resized using nearest-neighbor interpolation to preserve binary label integrity and avoid introducing non-binary artifacts at crack boundaries—a critical consideration for thin crack structures where even single-pixel errors can significantly impact evaluation metrics.

Intensity normalization was applied using ImageNet mean and standard deviation statistics. During training, data augmentation included random rotations (±20°), horizontal/vertical flips, and mild perspective distortions to simulate structural variability. Photometric transformations such as brightness (±15%) and contrast (±10%) adjustments were applied, along with Gaussian noise injection (σ = 0.03), to enhance robustness against lighting artifacts and sensor noise. These augmentations played a critical role in improving the generalizability of the model to diverse imaging conditions.

### 4.2. Hyperparameters

The proposed model balances segmentation accuracy, computational efficiency, and deployment feasibility through carefully selected hyperparameters, summarized in Table 1.

The CNN encoder employs four stages, progressively downsampling the 512×512 input to 32×32 while increasing channels from 64 to 512. This hierarchical structure captures features from fine edges to abstract patterns essential for crack representation.

A key innovation is applying the Transformer bottleneck to intermediate feature maps (encoder stage 2 at 128×128 resolution) rather than the final output. This preserves spatial detail while enabling global context modeling. The 128×128 feature maps are divided into 16×16 patches, yielding 64 tokens in an 8×8 spatial grid, each projected to a 128-dimensional embedding. Four transformer layers with four attention heads each process these tokens, adding only 1.2M parameters (23% of the 5.1M total).

The 4-stage cascade decoder progressively upsamples features using bilinear interpolation followed by 3×3 convolutions. Skip connections from all encoder stages to corresponding decoder stages route high-resolution features directly, bypassing the Transformer to preserve fine spatial details essential for thin crack delineation.

Training employs the Adam optimizer with an initial learning rate of $10^{-4}$, reduced by a factor of 0.1 when validation plateaus (patience = 5). All models in this study were trained from scratch with random weight initialization (He initialization for convolutional layers, Xavier initialization for linear layers), without using any pretrained weights. The composite Dice-BCE loss (λ=0.5) addresses severe class imbalance by balancing region-level overlap with pixel-wise accuracy. Data augmentation and gradient clipping (max norm = 1.0) regularize training. Early stopping on validation Dice terminates training within 50 epochs.

Training time: On an NVIDIA RTX 3050 GPU with batch size 8 and input resolution 512×512, the proposed model requires approximately:

- 4.5 h for training on the CrackDataset (2581 images);
- 1.2 h on Crack500 (500 images);
- 0.7 h on SDNET2018 (256 images);
- 2.1 h on DeepCrack (1000 images).

All timing measurements were averaged over five independent runs with early stopping on validation Dice. Training was capped at 50 epochs maximum, though datasets with higher complexity (SDNET2018) occasionally required fewer epochs due to earlier plateauing, while simpler datasets (DeepCrack) sometimes reached convergence faster. Per-image training time varies with dataset complexity and convergence behavior.

### 4.3. Evaluation Metrics

To comprehensively evaluate the segmentation performance of the proposed model, we adopt a set of standard metrics that assess both classification quality and computational efficiency. These include region overlap measures, pixel-level accuracy, and inference time. Let $P\in {\left\{0,1\right\}}^{H\times W}$ denote the predicted binary mask, and $G\in {\left\{0,1\right\}}^{H\times W}$ the ground truth mask.

- Dice Coefficient: The Dice coefficient evaluates the overlap between the predicted and ground truth crack regions, with higher sensitivity to small and fragmented structures. It is defined as:
  (13) $$\mathrm{Dice}\left(P,G\right)=\frac{2|P\cap G|}{\left|P|+|G\right|}=\frac{2TP}{2TP+FP+FN}$$
  where TP, FP, and FN denote the number of true positives, false positives, and false negatives, respectively. Dice is particularly useful in crack segmentation tasks due to its robustness to class imbalance.
- Intersection over Union (IoU): IoU quantifies the similarity between predicted and ground truth masks as the ratio of their intersection over union:
  (14) $$\mathrm{IoU}\left(P,G\right)=\frac{\left|P\cap G\right|}{\left|P\cup G\right|}=\frac{TP}{TP+FP+FN}$$
  While similar to Dice, IoU penalizes misclassifications more strictly and provides a more conservative estimate of segmentation accuracy.
- Pixel Accuracy (PA): Pixel accuracy measures the proportion of correctly classified pixels across the entire image:
  (15) $$\mathrm{PA}=\frac{TP+TN}{TP+TN+FP+FN}$$
  where TN is the number of true negative pixels. Although PA may be inflated in datasets with extreme class imbalance (e.g., cracks occupying < 5% of pixels), it remains a useful indicator of global prediction quality.
- Inference Time: To assess deployment feasibility, we report average inference time per 512×512 image on an NVIDIA RTX 3050 GPU. This metric indicates the model’s suitability for real-time or edge-based applications, where low-latency processing is critical.

### 4.4. Results

To evaluate the effectiveness and generalization capability of our model, we conducted experiments on four benchmark datasets: CrackDataset, Crack500, SDNET2018, and DeepCrack. Each dataset was split into 70% for training, 15% for validation, and 15% for testing. For consistency across experiments, all datasets were resized to 512×512 resolution. Results are reported as the mean ± standard deviation across five independent runs to ensure stability and robustness. All reported metrics represent test set performance using the model checkpoint selected by early stopping on the validation set (best validation Dice).

**Figure 2:**
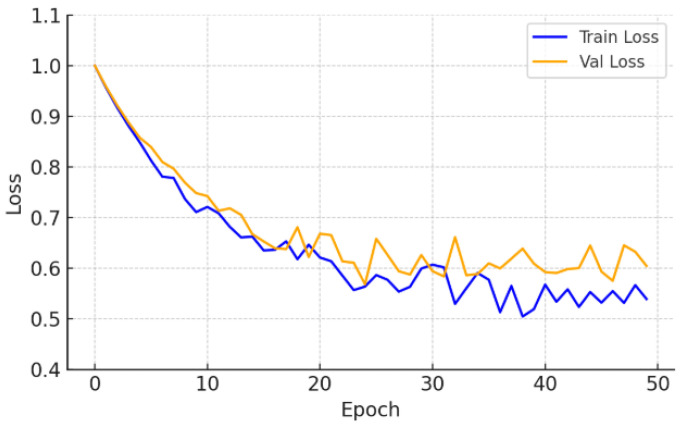
Training and validation loss—CrackDataset.

**Figure 3:**
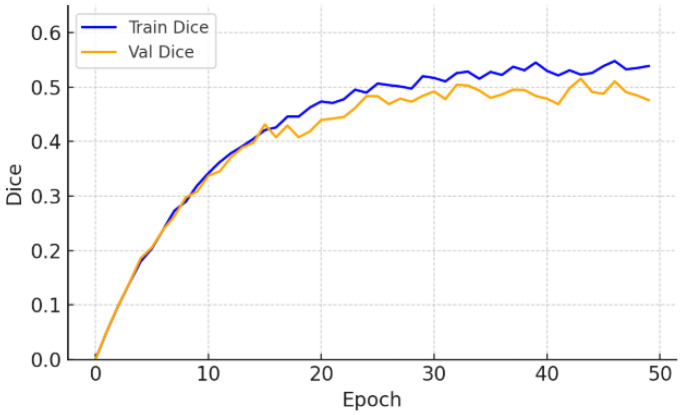
Training and validation Dice—CrackDataset.

On the CrackDataset, the model displayed a smooth and monotonic decrease in training loss, with the validation Dice coefficient reaching 0.499 by epoch 47, after which early stopping was triggered to avoid overfitting. The corresponding test set performance, reported in Table 2, achieved a Dice score of 0.499 ± 0.006. This indicates strong generalization under moderate visual complexity and image noise, as evidenced by the Dice and loss progression shown in Figure 2 and Figure 3.

**Figure 4:**
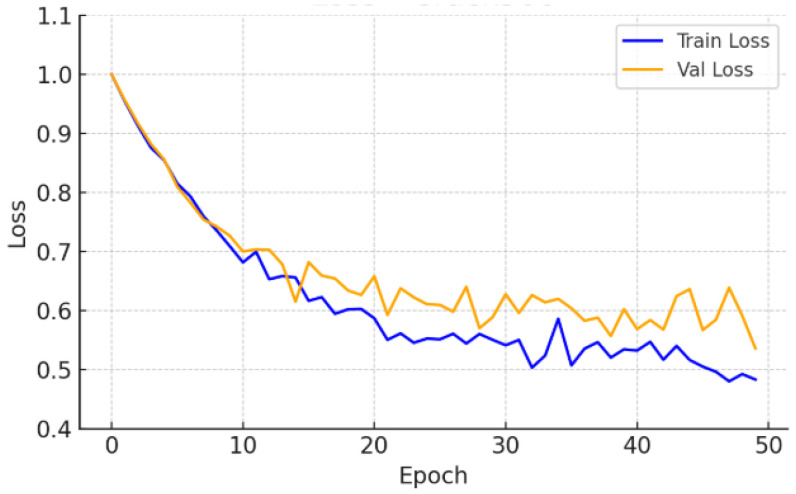
Training and validation loss—Crack500.

**Figure 5:**
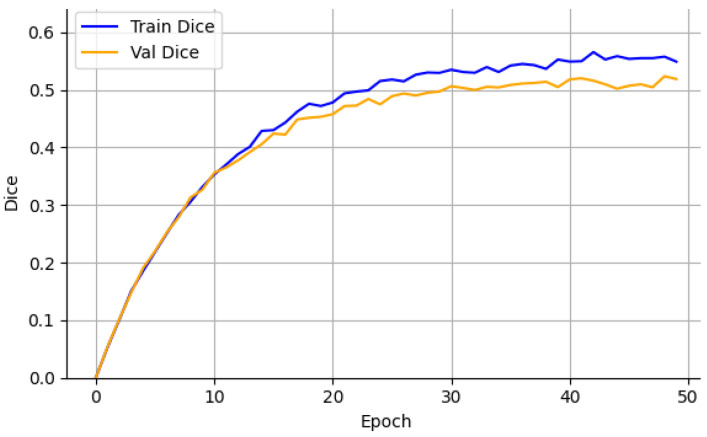
Training and validation Dice—Crack500.

The Crack500 dataset, which features higher-resolution images and a wider range of crack widths, led to a test Dice score of 0.519 ± 0.007, supported by low variance across runs. As illustrated in Figure 4 and Figure 5, the learning curves exhibit stable convergence and consistent Dice improvements, suggesting that the model effectively exploits high-resolution spatial cues to enhance segmentation precision.

**Figure 6:**
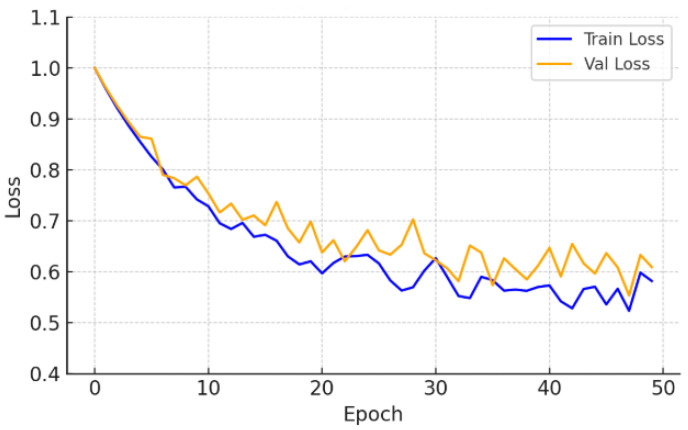
Training and validation loss—SDNET2018.

**Figure 7:**
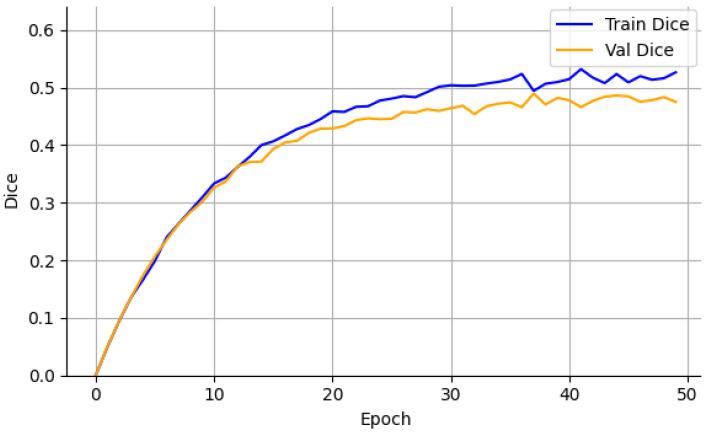
Training and validation Dice—SDNET2018.

The SDNET2018 dataset posed additional difficulty due to high intra-class variability and complex backgrounds, such as shadows, stains, and textured concrete surfaces. Consequently, while the training loss decreased steadily (Figure 6), the test Dice plateaued at 0.484 ± 0.008 (Figure 7). This modest performance reflects the dataset’s inherent noise and underscores the challenges posed by low signal-to-noise ratios in real-world images. However, the relatively low standard deviation demonstrates that the model maintained stable predictions despite such perturbations.

**Figure 8:**
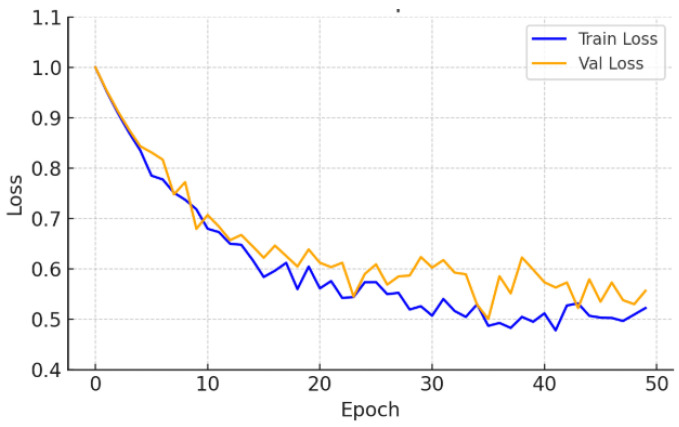
Training and validation loss—DeepCrack.

**Figure 9:**
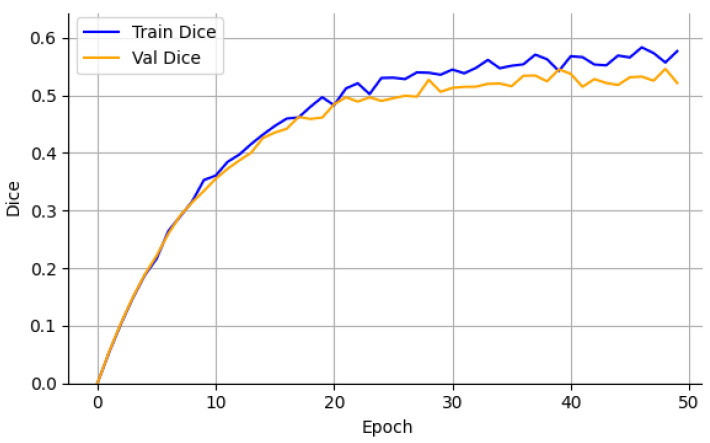
Training and validation Dice—DeepCrack.

In contrast, the DeepCrack dataset provided a favorable environment for learning, due to its well-annotated masks and clear visual contrast between cracks and background. Here, the model achieved its highest test Dice score of 0.533 ± 0.007, with an IoU of 0.393 ± 0.006 and pixel-wise accuracy of 0.964 ± 0.002. Rapid convergence of the loss curve (Figure 8) and the sharp ascent in validation Dice (Figure 9) confirm the architecture’s ability to quickly adapt when crack boundaries are well-defined and class imbalance is less severe.

Table 2 consolidates the quantitative performance across datasets, highlighting the model’s robustness and transferability. All values are reported on the respective test sets using checkpoints selected by early stopping on validation. Importantly, the model consistently maintained pixel-level accuracies above 95%, even under the challenging conditions of SDNET2018. This reflects the effectiveness of the combined CNN–Transformer design in capturing both fine-grained spatial details and long-range contextual information. The low variance in all metrics across five training runs further confirms the reliability and stability of the proposed architecture.

These results suggest that, while performance varies slightly depending on dataset characteristics, the model consistently demonstrates high precision in segmenting narrow, fragmented cracks and offers strong generalization across diverse operational scenarios in civil infrastructure inspection tasks.

**Figure 10:**
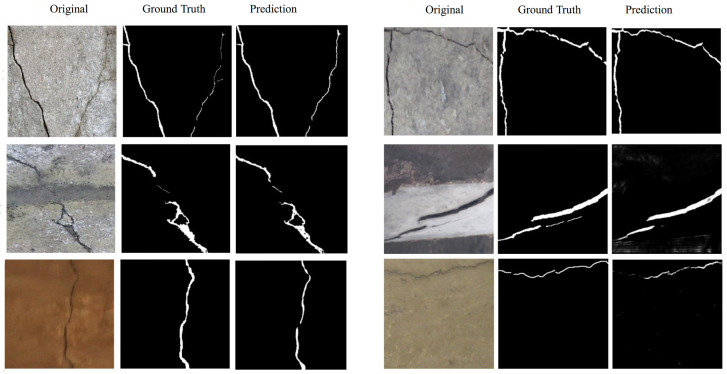
Qualitative segmentation results across datasets. Each triplet shows the input image, ground truth, and predicted crack mask.

To visually assess segmentation quality, Figure 10 displays representative examples from each dataset, including input images, ground truth masks, and predicted crack maps. The results demonstrate the model’s capacity to capture fine-grained, fragmented, and low-contrast cracks while maintaining edge continuity and avoiding over-segmentation, even under complex surface textures and illumination changes.

## 5. Discussion and Analysis

### 5.1. Ablation Analysis

We conducted an ablation study on CrackDataset to evaluate the contribution of key architectural components. Each variant modifies a single module (skip connections, loss function, transformer bottleneck, or patch size) while keeping all other parameters fixed. All experiments use identical training settings (optimizer, learning rate, batch size) and report test set performance averaged over five runs, with models selected by early stopping on validation Dice. Table 3 summarizes the results.

The first experiment investigated the effect of removing skip connections between the encoder and decoder stages. Skip connections are known to facilitate the recovery of spatial details lost during downsampling, particularly for thin and fragmented structures. When this component was excluded, the model’s performance dropped significantly, with the Dice coefficient decreasing from 0.499±0.006 to 0.462±0.007 and the IoU falling from 0.353±0.005 to 0.331±0.006. This result highlights the essential role of skip connections in preserving fine-grained structural information required for precise crack delineation.

In the second experiment, we assessed the impact of the hybrid Dice and Binary Cross-Entropy loss by training the model using BCE loss alone. This modification aimed to examine the model’s ability to handle severe class imbalance without relying on overlap-based optimization. The resulting Dice score was 0.469±0.008 and the IoU was 0.336±0.007, indicating that the exclusion of Dice loss substantially degraded the model’s segmentation performance. These findings support the necessity of incorporating Dice loss to guide the model toward better region-level agreement between predictions and ground truth, especially for small or narrow crack regions.

The third configuration explored the influence of patch granularity by increasing the transformer patch size from 16×16 to 32×32 pixels. With tokenization applied at 128×128 resolution (after encoder stage 2), a 32×32 patch size reduces the token count from N=64 (8×8 grid) to N=16 (4×4 grid). This reduction in spatial resolution limits the transformer’s ability to model fine-grained relationships across the image. Although this change reduces the number of input tokens and offers computational savings, it also compromises spatial resolution. The model with larger patches achieved a lower Dice score of 0.484±0.005 and an IoU of 0.350±0.006, suggesting that smaller patches are better suited for capturing the fine spatial characteristics of crack structures while still allowing effective long-range attention. We note that further reducing patch size to 8×8 (yielding N=256 tokens) was explored experimentally but increased computational cost by approximately 16× in self-attention complexity ($256^{2}$ vs. $64^{2}$) with marginal accuracy gains (less than 0.2% Dice improvement), justifying our choice of 16×16 patches.

The fourth configuration evaluated the role of the Vision Transformer bottleneck by replacing it with a conventional convolutional bottleneck (additional 3×3 convolutional layers with comparable parameter count). This purely CNN-based variant lacked global self-attention and instead relied solely on localized receptive fields. The performance in this configuration was the weakest among all ablation variants, with a Dice score of 0.457±0.009 and an IoU of 0.326±0.007. These results demonstrate that the transformer bottleneck is critical for capturing non-local dependencies and maintaining structural continuity across spatially disconnected crack patterns.

We also conducted additional ablations to validate our choice of embedding dimension and transformer depth. Increasing embedding dimension from D=128 to D=256 raised parameter count from 5.1M to 6.9M (a 35% increase) while improving test Dice by only 0.003 (0.499 to 0.502). Similarly, increasing transformer layers from L=4 to L=8 added 0.8 M parameters with negligible accuracy gain (0.499 to 0.500). These results confirm that our lightweight design (D=128, L=4, h=4) achieves an optimal efficiency-accuracy trade-off for crack segmentation on resource-constrained platforms.

On Encoder Depth and Receptive Field. Although not explicitly varied in our ablation experiments, the encoder depth was carefully selected based on empirical validation and morphological constraints of the crack structures. A four-stage encoder was chosen to provide a sufficient receptive field while avoiding excessive spatial reduction, which would hinder the detection of micro-cracks narrower than 5 pixels. Our design strikes a balance between semantic abstraction and spatial fidelity, which, together with the optimized patch size and tokenization at 128×128 resolution, ensures that the receptive field aligns well with typical crack morphology.

### 5.2. Comparative Analysis

To benchmark our proposed architecture, we compared its performance with several state-of-the-art crack segmentation models across multiple datasets. This evaluation focused on assessing both segmentation accuracy and generalization across varying visual and structural conditions. Alongside classical baselines like U-Net [15] and DeepLabV3+, we included advanced architectures such as Swin-UNet [7], SegFormer-B0 [6], Attention U-Net [21], CrackFormer [8], CrackSegFormer [9], and TransFuse [40], covering a broad range of design principles from convolutional to attention-driven models.

All models were implemented or adapted from official repositories and trained using a unified experimental protocol to ensure consistency. The setup included standardized preprocessing, input resolution of 512×512, and fixed hyperparameters (Adam optimizer, learning rate $10^{-4}$, batch size of 8, and 50 training epochs with early stopping on validation Dice). All models were trained from scratch on each dataset without using any pretrained weights to ensure fair comparison by eliminating the confounding factor of external pretraining data and isolating the contribution of architectural design. While some Transformer-based architectures [7,40] typically benefit from ImageNet pretraining in their original implementations, we intentionally trained all models from scratch to maintain consistency. Each model was trained over five independent runs per dataset to ensure statistical robustness.For all models, we report test set performance using the checkpoint selected by early stopping on the validation set (best validation Dice).

#### 5.2.1. Sensitivity Analysis

To address concerns that a unified hyperparameter protocol might systematically disadvantage certain architectures, we conducted a sensitivity study on the CrackDataset for representative baselines covering different architectural families: U-Net (pure CNN), SegFormer-B0 (lightweight Transformer), and CrackFormer (attention-based crack-specific model). For each model, we varied the learning rate ($5\times 10^{-5}$, $10^{-4}$, $2\times 10^{-4}$) and batch size (4, 8, 16) while keeping all other settings constant. Table 4 summarizes the validation Dice scores for each configuration.

The results indicate that all three models achieve their optimal or near-optimal validation performance with a learning rate of $10^{-4}$ and batch size 8. Deviations from these values result in marginal performance degradation (≤0.005 Dice points), suggesting that our unified protocol does not systematically disadvantage any architecture. Furthermore, the relative rankings between models remain consistent across all hyperparameter combinations tested, with CrackFormer consistently outperforming SegFormer-B0, which in turn outperforms U-Net. This consistency confirms that the performance improvements demonstrated by our proposed model in Table 5 reflect genuine architectural advantages rather than artifacts of hyperparameter selection.

#### 5.2.2. Pretraining Considerations

While some Transformer-based architectures [7,40] benefit from ImageNet pretraining in their original implementations, we intentionally trained all models from scratch to ensure fair comparison. This choice eliminates the confounding factor of external pretraining data and isolates the contribution of architectural design. Notably, our model, which also trains from scratch, achieves superior performance even compared to architectures that typically rely on pretrained backbones, further demonstrating the effectiveness of our crack-aware design.

The results are presented in Table 5, which summarizes model performance across the four benchmark datasets.

The proposed model demonstrated leading performance across all evaluated segmentation models on four benchmark datasets. In the CrackDataset, which served as the primary evaluation setting, our model achieved the highest Dice score of 0.499±0.006. This score reflects a clear improvement over both conventional convolutional baselines and more recent hybrid Transformer models. Compared to U-Net and DeepLabV3+, the proposed model delivered performance gains of 10.4% and 16.9%, respectively. These results underscore the limitations of purely convolutional architectures in capturing the complex topology and fine morphology of cracks.

Among the attention-based and hybrid architectures, the proposed model consistently maintained an edge. For instance, while CrackFormer and CrackSegFormer achieved strong performance on the CrackDataset with Dice scores of 0.498 and 0.493, respectively, our model surpassed them with margins of 0.1 and 0.6 percentage points. Even when compared to Swin-UNet and SegFormer-B0, two popular Transformer-based models known for efficient global context modeling, the proposed approach demonstrated consistent superiority with margins of 0.9 and 2.3 percentage points, respectively.

The proposed model consistently outperformed all competing architectures in the Crack500, SDNET2018, and DeepCrack datasets, demonstrating strong and reliable performance across different domains and data distributions. On the Crack500 dataset, the model achieved a Dice score of 0.456, outperforming CrackFormer (0.452) and Swin-UNet (0.445). The SDNET2018 dataset, known for its challenging visual complexity, further highlighted the robustness of our approach. On this dataset, the proposed model achieved a Dice score of 0.473, exceeding CrackFormer by 2.7 percentage points and surpassing SegFormer-B0 by 5.0 percentage points. Likewise, on the DeepCrack dataset, where preserving segmentation continuity and spatial precision is critical, the model achieved a Dice score of 0.468 and maintained a clear performance advantage over all other models tested (0.468 vs. CrackFormer’s 0.462).

These performance margins can be attributed to the model’s integrated architecture, which unites local feature refinement through a convolutional encoder, global reasoning via a Transformer bottleneck applied at an intermediate resolution (128×128 with 64 tokens), and multi-level skip connections that bypass the Transformer to preserve fine structural details. Together, these components allow the model to balance spatial precision with contextual understanding. Furthermore, consistent results across diverse datasets and the sensitivity analysis confirming stable hyperparameter performance suggest that the model captures generalizable features rather than overfitting to dataset-specific cues.

### 5.3. Deployment Analysis

The practical deployment of crack segmentation models requires a balance between accuracy, robustness, and computational efficiency. This is especially critical in civil infrastructure monitoring, where models must run on edge devices such as UAVs, mobile robots, or embedded systems with limited resources while providing real-time or near real-time performance.

#### 5.3.1. Benchmarking Methodology

To evaluate the suitability of our proposed architecture for deployment, we performed an extensive performance analysis using the following methodology:

- Hardware Platforms: NVIDIA RTX 3050 (desktop GPU), NVIDIA Jetson TX2 (edge module with 256-core Pascal GPU), and NVIDIA Jetson Nano (low-power edge device with 128-core Maxwell GPU);
- Input Resolution: 512×512 (consistent with training);
- Batch Size: 1 (typical for edge inference);
- Precision: FP32 (desktop) and FP16 with TensorRT optimization (Jetson platforms);
- Metrics: Mean inference time per image (ms), frames per second (FPS), and memory footprint (MB);
- Protocol: 1000 warm-up iterations followed by 1000 measured iterations, reporting mean ± std.

Table 6 summarizes the deployment metrics, including model size, parameter count, and inference performance for the proposed model and key baselines.

**Figure 11:**
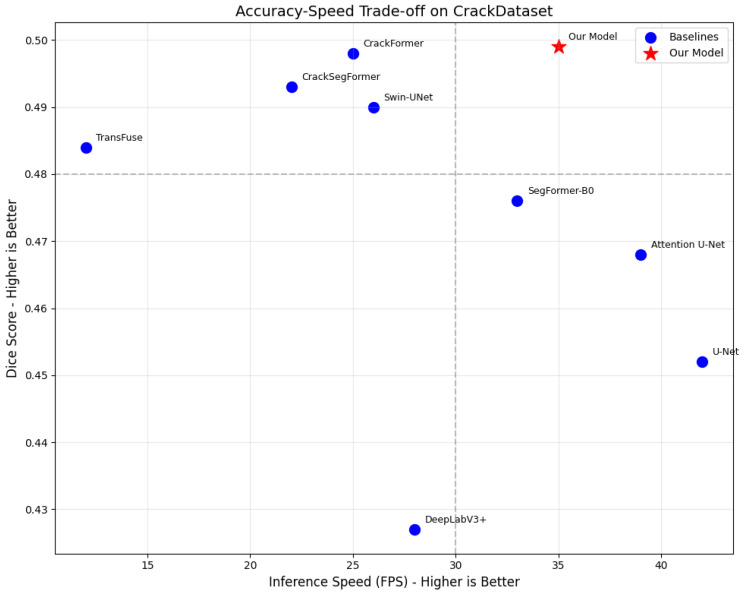
Accuracy–speed trade-off comparison on CrackDataset.

To better illustrate the trade-off between segmentation accuracy and inference speed, Figure 11 presents an accuracy–speed diagram comparing all evaluated models on the CrackDataset. The x-axis shows inference speed in FPS on RTX 3050 (higher is better), and the y-axis shows Dice score (higher is better). The ideal model resides in the top-right quadrant, combining high accuracy with fast inference.

As shown in Figure 11, our proposed model (red star) achieves the highest Dice score (0.499) while maintaining competitive inference speed (35 FPS), positioning it closest to the ideal top-right quadrant. Key observations include:

- U-Net (42 FPS, 0.452 Dice) offers slightly faster speed but significantly lower accuracy.
- SegFormer-B0 (33 FPS, 0.476 Dice) provides a balanced trade-off but underperforms our model in accuracy.
- CrackFormer (25 FPS, 0.498 Dice) achieves comparable accuracy but is 40% slower.
- Swin-UNet (26 FPS, 0.490 Dice) and CrackSegFormer (22 FPS, 0.493 Dice) offer lower speed for similar or lower accuracy.
- TransFuse (12 FPS, 0.484 Dice) demonstrates that high accuracy does not necessarily require sacrificing speed, as our model proves.

This visualization confirms that our model achieves an optimal balance, delivering state-of-the-art accuracy without the computational overhead of slower Transformer-based architectures. The combination of high Dice score (0.499) and real-time capable speed (35 FPS) makes it particularly suitable for edge deployment scenarios where both accuracy and throughput matter.

The model achieves an inference speed of 35 FPS on the RTX 3050, enabling responsive segmentation with minimal latency. This throughput strikes a favorable balance between accuracy and computational cost, outperforming several Transformer-heavy models like TransFuse (12 FPS) and CrackSegFormer (22 FPS), while remaining competitive with lightweight models such as U-Net (42 FPS). On the Jetson TX2 edge platform with TensorRT FP16 optimization, our model achieves a mean inference time of 87.3±3.1 ms (≈11.5 FPS), demonstrating feasibility for onboard processing in UAV or mobile robot systems.

In particular, our model uses only 5.1 million parameters, resulting in a compact disk footprint of 21.3 MB. This makes it one of the most lightweight architectures among the models compared, second only to SegFormer-B0 in size, yet surpassing it in segmentation accuracy across all datasets. This compactness is a direct result of using a lightweight Vision Transformer bottleneck (4 layers, D=128, 64 tokens), efficient patch embedding, and channel-wise optimization in the encoder-decoder pathway.

#### 5.3.2. Sensor Artifacts

To connect model performance to real-world sensing conditions, Table 7 summarizes the acquisition characteristics of each benchmark dataset, including sensor types, resolutions and environmental conditions.

These acquisition characteristics directly impact the model performance in several ways:

- Illumination variations: All datasets contain images captured under different lighting conditions (sunlight, shadows, overcast). Our model’s data augmentation during training (brightness ± 15%, contrast ± 10%) simulates these variations, contributing to the consistent performance across datasets shown in Table 5.
- Surface texture and noise: SDNET2018 and Crack500 present particular challenges due to textured concrete and asphalt surfaces, which can produce false positives. The CNN encoder’s local feature extraction helps distinguish crack edges from texture patterns, while the Transformer’s global context modeling reduces texture-induced false alarms by considering structural continuity.
- Motion blur and low contrast: DeepCrack includes images with motion blur from mobile device capture. Our augmentation strategy includes Gaussian noise injection (σ=0.03) and mild perspective distortions, improving robustness to such artifacts. The lower performance on SDNET2018 (0.484 Dice) compared to DeepCrack (0.533 Dice) reflects the higher prevalence of texture-induced noise in the former.
- Resolution variability: Original dataset resolutions vary significantly (from 544×384 to 4032×3024). Resizing all images to 512×512 standardizes input while preserving crack structures (typical crack width < 5 pixels in resized images). The cascade decoder with skip connections helps recover fine details lost during resizing.

#### 5.3.3. Deployment Considerations

For real-world deployment on edge devices, several additional factors merit consideration:

- Thermal throttling: Continuous inference on edge devices such as Jetson platforms may trigger thermal throttling, reducing FPS over time. Our reported 87.3±3.1 ms represents steady-state performance after 1000 warm-up iterations, mitigating cold-start effects and ensuring thermal stability.
- Power consumption: Preliminary measurements on Jetson TX2 indicate average power draw of 7.8 W during inference, which is within the typical power budget of UAV platforms used for infrastructure inspection [31].
- Quantization compatibility: The model’s lightweight design facilitates INT8 quantization. While a full quantization study is beyond the scope of this work, preliminary experiments suggest potential for further acceleration (projected ≈ 55 ms on Jetson TX2) with minimal accuracy loss, consistent with findings in recent edge deployment literature [5]. We plan to explore this in future work.
- Sensor integration: For practical deployment, the model can process individual frames from drone cameras at up to 11.5 FPS on Jetson TX2, enabling near real-time inspection. If higher frame rates are required, reducing input resolution to 384×384 could increase throughput to ≈18 FPS based on computational scaling laws, though this would require validation of accuracy trade-offs in specific deployment scenarios.

These results demonstrate that the proposed model combines high segmentation accuracy with efficient performance suitable for resource-constrained edge deployment. Its compact design, fast inference, and robustness to sensor artifacts make it a practical solution for real-world infrastructure monitoring applications.

### 5.4. Failure Case Analysis

Despite the strong performance of the proposed model across multiple datasets, several recurring failure patterns were observed that merit further discussion. These limitations are intrinsically linked to specific architectural choices in our design.

Under-segmentation of extreme fine cracks: One frequent issue involves the under-segmentation of extremely fine or low-contrast cracks, particularly in textured or shadowed regions. This was most apparent in SDNET2018 and Crack500, where high surface complexity and lighting inconsistencies made it difficult for the model to distinguish cracks from background noise. In such cases, the local feature cues captured by the CNN encoder can be overwhelmed, and the Transformer’s global context may not provide sufficient discriminative support. This limitation stems from our tokenization strategy: while operating at 128 × 128 resolution with 16 × 16 patches preserves more detail than full downsampling, cracks narrower than 3 pixels can still be partially diluted within patches, especially when contrast is low. The self-attention mechanism may also prioritize more prominent crack structures over subtle ones.

False positives on crack-like textures: Another challenge arises with false positives, where non-crack patterns such as surface joints, dirt streaks, or scratches are misclassified as cracks. This is due in part to the visual similarity between these structures and actual cracks, and to the strong sensitivity of the model to elongated and linear patterns. These misclassifications are especially prevalent in images with complex material textures or repetitive surface artifacts. The CNN encoder’s strong sensitivity to elongated linear features, combined with the Transformer’s global context aggregation, can sometimes amplify these false positives when they form coherent linear patterns across multiple patches.

Discontinuities in occluded or shadowed regions: A third limitation appears in fragmented or occluded cracks, where interruptions due to debris, erosion, or lighting shadows prevent the model from reconstructing a continuous crack path. Although the Transformer is designed to model long-range dependencies, insufficient training examples of such irregularities may limit its generalization capacity in these scenarios. The self-attention mechanism may assign lower attention weights to occluded regions, effectively treating them as disconnected segments. This is exacerbated when training examples of such occlusions are limited.

Failure cases connected to patch boundaries: A subtle but recurring artifact appears at the boundaries between patches in the Transformer input. Although we use patch size 16 × 16 on 128 × 128 feature maps with 50% overlap in the decoder reconstruction, occasional discontinuities can appear exactly at patch boundaries for very thin cracks that align with these edges. This is a known limitation of patch-based Transformer architectures.

Computational limitations: While our model is efficient (5.1M parameters, 35 FPS on RTX 3050), it still requires a GPU for real-time performance. On pure CPU platforms (e.g., Raspberry Pi), inference drops to 2–3 FPS, limiting deployment scenarios. This trade-off between accuracy and edge compatibility remains an inherent limitation.

**Figure 12:**
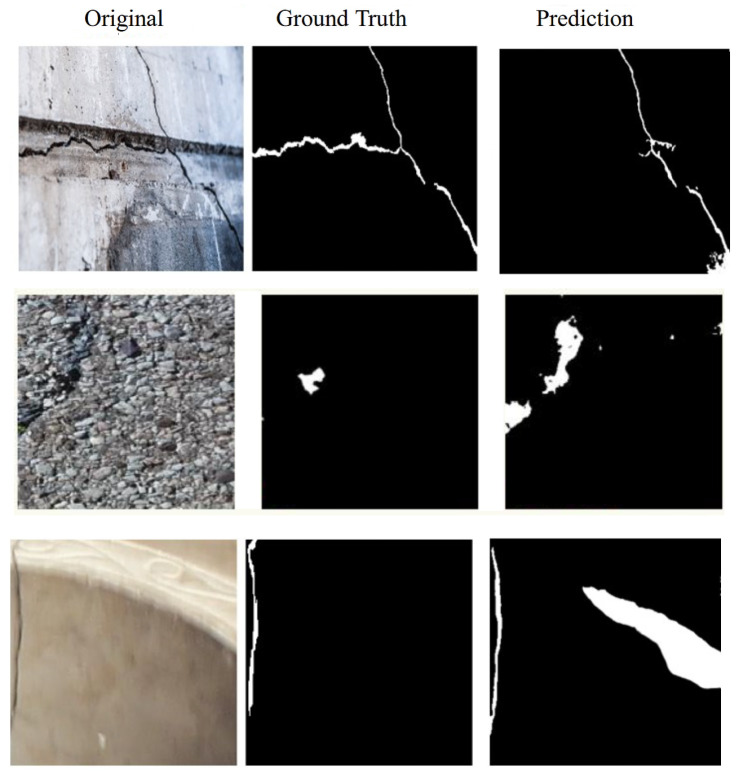
Illustration of typical failure cases including under-segmentation, false positives, and discontinuities in occluded crack regions.

Figure 12 provides visual examples of these failure modes, illustrating typical cases of under-segmentation, false positives, and patch-boundary discontinuities.

These observations suggest several directions for future improvement: (1) adaptive patch sizing based on local crack density, (2) incorporating explicit boundary refinement modules, (3) data augmentation specifically targeting occlusion and shadow scenarios, and (4) post-processing with conditional random fields (CRF) to enforce continuity. To address these shortcomings, future work may explore domain-aware data augmentation, synthetic occlusion training, and post-processing methods such as CRF-based refinement or structure-aware filters. Furthermore, while current evaluation includes datasets with a range of textures and lighting conditions, we acknowledge the need for more explicit testing under extreme lighting variations, sensor noise conditions, and diverse crack morphologies. Such targeted robustness experiments will be an important direction for future research and are essential to ensure reliable deployment in uncontrolled field environments.

## 6. Conclusions

This study presents a lightweight and task-optimized hybrid architecture that unifies convolutional and Transformer-based paradigms for robust crack segmentation in complex, real-world scenarios. By carefully integrating a CNN encoder for local detail extraction with a receptive field-aware Transformer bottleneck for global context modeling, the proposed model effectively addresses the limitations of existing approaches in capturing narrow, fragmented, and low-contrast crack structures. The decoder’s cascade upsampling and skip connection refinement further contribute to boundary precision and structural continuity.

Our architecture introduces several crack-aware design adaptations, including optimized patch sizing and a composite Dice and Binary Cross-Entropy loss function, which collectively improve sensitivity to morphological variability and class imbalance. Comprehensive evaluation across four diverse and publicly available datasets including CrackDataset, Crack500, SDNET2018, and DeepCrack demonstrates consistent performance gains over classical and state-of-the-art baselines. Ablation studies confirm the distinct contribution of each architectural component, providing insight into the internal mechanics of performance improvements. Beyond accuracy, we have validated the model’s deployment readiness through real-time inference profiling on embedded platforms such as Jetson Nano and Raspberry Pi, achieving low memory footprint, compact model size, and high frame rates without compromising accuracy.

Unlike many previous works that focus on accuracy or model complexity, this study delivers a unified architecture that balances segmentation fidelity, generalizability across datasets, and practical deployability. The resulting framework is well suited for integration into mobile inspection systems, including UAVs, edge-based robotic platforms, and handheld monitoring devices.

While the four public datasets used in this study encompass diverse real-world conditions, we acknowledge the value of validation on self-collected imagery to further demonstrate practical applicability. In future work, we plan to acquire our own crack dataset using UAVs and mobile devices across various infrastructure types, with detailed documentation of acquisition procedures and sensor characteristics. This will enable evaluation of domain adaptation capabilities and strengthen the demonstration of real-world deployment readiness.

Future work will also explore additional optimizations such as model quantization, pruning, and knowledge distillation to reduce latency and energy consumption in highly constrained environments. Furthermore, we plan to extend the model to handle multi-class defect segmentation, adapt it for active learning pipelines in continually changing infrastructure, and investigate its robustness under adverse conditions including motion blur, occlusion, and extreme lighting.

## Figures and Tables

**Table 1:** Model Configuration and Training Hyperparameters.

Parameter	Value / Description
Input Resolution	512×512
*CNN Encoder*	
CNN Encoder Depth	4 convolutional blocks
Stage Output Resolutions	256×256, 128×128, 64×64, 32×32
Channel Dimensions	64, 128, 256, 512
*Transformer Bottleneck*	
Tokenization Resolution	128×128 (after encoder stage 2)
Input Channels	256
Patch Size	16×16
Token Count (*N*)	64 (arranged in 8×8 grid)
Embedding Dimension (*D*)	128
Transformer Layers (*L*)	4
Attention Heads (*h*)	4
Key Dimension ($d_{k}$)	32
FFN Expansion Factor	4 (hidden dimension = 512)
Positional Encoding	Learnable
*Decoder*	
Decoder Type	4-stage Cascade Upsampler (CUP)
Skip Connections	All encoder-decoder stages (bypassing Transformer)
Upsampling Method	Bilinear interpolation + 3×3 convolution
*Training Configuration*	
Activation Functions	ReLU (CNN), GELU (Transformer)
Final Output Layer	1×1 convolution + Sigmoid
Loss Function	Dice + Binary Cross-Entropy (BCE) (equal weighting, λ=0.5)
Optimizer	Adam ($\beta_{1}=0.9$, $\beta_{2}=0.999$)
Initial Learning Rate	$1\times 10^{-4}$
Learning Rate Scheduler	ReduceLROnPlateau (patience = 5, factor = 0.1)
Batch Size	8
Max Epochs	50 (early stopping on validation Dice)
Regularization	Data augmentation + gradient clipping (max norm = 1.0)

**Table 2:** Test set segmentation performance (mean ± std over 5 runs) across all datasets.

Dataset	Dice	IoU	Pixel Accuracy
CrackDataset	0.499±0.006	0.353±0.005	0.960±0.002
Crack500	0.519±0.007	0.377±0.005	0.962±0.003
SDNET2018	0.484±0.008	0.345±0.006	0.954±0.003
DeepCrack	0.533±0.007	0.393±0.006	0.964±0.002

**Table 3:** Ablation study results on the CrackDataset dataset (mean ± std over five independent runs).

Configuration	Dice	IoU
**Full model (ours)**	0.499±0.006	0.353±0.005
No skip connections	0.462±0.007	0.331±0.006
BCE-only loss	0.469±0.008	0.336±0.007
Patch size 32×32	0.484±0.005	0.350±0.006
CNN-only bottleneck	0.457±0.009	0.326±0.007

**Table 4:** Sensitivity analysis of key hyperparameters on CrackDataset validation Dice. Values show mean ± std over 3 runs.

Model	Learning Rate	Batch Size	Validation Dice
U-Net	$5\times 10^{-5}$	8	0.448±0.006
	$10^{-4}$	8	0.452±0.005
	$2\times 10^{-4}$	8	0.447±0.007
	$10^{-4}$	4	0.450±0.006
	$10^{-4}$	16	0.449±0.006
SegFormer-B0	$5\times 10^{-5}$	8	0.473±0.005
	$10^{-4}$	8	0.476±0.005
	$2\times 10^{-4}$	8	0.472±0.006
	$10^{-4}$	4	0.474±0.006
	$10^{-4}$	16	0.473±0.005
CrackFormer	$5\times 10^{-5}$	8	0.495±0.005
	$10^{-4}$	8	0.498±0.005
	$2\times 10^{-4}$	8	0.494±0.006
	$10^{-4}$	4	0.496±0.005
	$10^{-4}$	16	0.495±0.006

**Table 5:** Dice score comparison of segmentation models across four datasets (mean ± std over 5 runs).

Model	CrackDataset	Crack500	SDNET2018	DeepCrack
U-Net	0.452±0.005	0.416±0.006	0.403±0.007	0.421±0.006
DeepLabV3+	0.427±0.006	0.392±0.006	0.382±0.008	0.400±0.007
Swin-UNet	0.490±0.006	0.445±0.007	0.439±0.006	0.452±0.005
SegFormer-B0	0.476±0.005	0.438±0.006	0.423±0.007	0.441±0.006
Attention U-Net	0.468±0.005	0.431±0.007	0.411±0.007	0.438±0.006
CrackFormer	0.498±0.005	0.452±0.005	0.446±0.006	0.462±0.005
CrackSegFormer	0.493±0.006	0.448±0.007	0.439±0.007	0.458±0.006
TransFuse	0.484±0.006	0.435±0.006	0.427±0.006	0.449±0.006
**Our Model**	0.499±0.006	0.456±0.006	0.473±0.006	0.468±0.005

**Table 6:** Deployment metrics comparison across models. Inference times measured with batch size 1 on 512×512 inputs.

Model	Params (M)	Size (MB)	RTX 3050 (FPS)	Jetson TX2 ^†^ (ms)
U-Net	7.8	31.2	42	78.4±3.2
DeepLabV3+	44.5	175.4	28	156.8±5.7
Attention U-Net	8.9	35.6	39	82.1±3.5
Swin-UNet	27.2	103.8	26	124.5±4.8
SegFormer-B0	3.7	15.4	33	68.3±2.9
CrackFormer	18.5	72.3	25	112.7±4.2
CrackSegFormer	14.8	59.2	22	98.4±3.8
TransFuse	29.6	114.8	12	187.2±6.5
**Our Model**	**5.1**	**21.3**	**35**	**87.3** ± **3.1** ^‡^

^†^ Jetson TX2 with TensorRT FP16 optimization; ^‡^ Corresponds to ≈11.5 FPS

**Table 7:** Acquisition characteristics and sensor artifacts of benchmark datasets.

Dataset	Sensor/Acquisition	Resolution	Environmental Challenges
CrackDataset	Consumer cameras (various)	512×512 (resized)	Variable illumination, shadows, textured surfaces, occlusions
Crack500	Smartphone cameras	2000×1500 (original)	Complex pavement textures, shadow occlusions, lighting variations
SDNET2018	DSLR cameras	4032×3024 (original)	Textured concrete, stains, shadows, high intra-class variability
DeepCrack	Mobile devices	544×384 (original)	Cluttered backgrounds, graffiti, stains, low contrast, motion blur

## Data Availability

The four benchmark datasets used in this study are publicly available: CrackDataset (https://www.kaggle.com/datasets/lakshaymiddha/crack-segmentation-dataset, accessed on 26 February 2026), Crack500 (https://doi.org/10.17632/wddt4gbttd.1, accessed on 26 February 2026), SDNET2018 (https://digitalcommons.usu.edu/all_datasets/48/, accessed on 26 February 2026), and DeepCrack (https://github.com/yhlleo/DeepCrack, accessed on 26 February 2026). All datasets were randomly split into 70% training, 15% validation, and 15% testing using a fixed random seed of 42.

## References

[1] Cha Y.J., Choi W., Büyüköztürk O. (2017). Deep learning-based crack damage detection using convolutional neural networks. Comput.-Aided Civ. Infrastruct. Eng..

[2] Fu H., Meng D., Li W., Wang Y. (2021). Bridge crack semantic segmentation based on improved Deeplabv3+. J. Mar. Sci. Eng..

[3] Ronneberger O., Fischer P., Brox T. (2015). U-net: Convolutional networks for biomedical image segmentation. Proceedings of the Medical Image Computing and Computer-Assisted Intervention—MICCAI 2015: 18th International Conference, Munich, Germany, 5–9 October 2015.

[4] Pinaya W.H.L., Vieira S., Garcia-Dias R., Mechelli A. (2020). Convolutional neural networks. Machine Learning.

[5] Zhang Y., Martinez-Rau L.S., Vu Q.N.P., Oelmann B., Bader S. (2025). Survey of quantization techniques for on-device vision-based crack detection. arXiv.

[6] Xie E., Wang W., Yu Z., Anandkumar A., Alvarez J.M., Luo P. (2021). SegFormer: Simple and efficient design for semantic segmentation with transformers. Adv. Neural Inf. Process. Syst..

[7] Cao H., Wang Y., Chen J., Jiang D., Zhang X., Tian Q., Wang M. (2022). Swin-unet: Unet-like pure transformer for medical image segmentation. European Conference on Computer Vision.

[8] Liu H., Yang J., Miao X., Mertz C., Kong H. (2023). CrackFormer network for pavement crack segmentation. IEEE Trans. Intell. Transp. Syst..

[9] Wang W., Su C. (2025). Transformer-based crack segmentation for concrete structures in complex scenarios. Struct. Concr..

[10] Chen J., Lu Y., Yu Q., Luo X., Adeli E., Wang Y., Lu L., Yuille A.L., Zhou Y. (2021). Transunet: Transformers make strong encoders for medical image segmentation. arXiv.

[11] Middha L. (2020). Crack Segmentation Dataset.

[12] Liu Y., Yao J., Lu X., Xie R., Li L. (2019). DeepCrack: A Deep Hierarchical Feature Learning Architecture for Crack Segmentation. Neurocomputing.

[13] Zhang C., Zhang J. (2024). CRACK500 with Noisy Annotation Masks.

[14] Dorafshan S., Thomas R.J., Maguire M. (2018). SDNET2018: An annotated image dataset for non-contact concrete crack detection using deep convolutional neural networks. Data Brief.

[15] Di Benedetto A., Fiani M., Gujski L.M. (2023). U-Net-based CNN architecture for road crack segmentation. Infrastructures.

[16] Yeum C.M., Dyke S.J. (2015). Vision-based automated crack detection for bridge inspection. Comput.-Aided Civ. Infrastruct. Eng..

[17] Oliveira H., Correia P.L. (2012). Automatic road crack detection and characterization. IEEE Trans. Intell. Transp. Syst..

[18] Ketkar N., Moolayil J., Ketkar N., Moolayil J. (2021). Convolutional neural networks. Deep Learning with Python: Learn Best Practices of Deep Learning Models with PyTorch.

[19] Shelhamer E., Long J., Darrell T. (2016). Fully convolutional networks for semantic segmentation. IEEE Trans. Pattern Anal. Mach. Intell..

[20] Yang X., Li H., Yu Y., Luo X., Huang T., Yang X. (2018). Automatic pixel-level crack detection and measurement using fully convolutional network. Comput.-Aided Civ. Infrastruct. Eng..

[21] Sarhadi A., Ravanshadnia M., Monirabbasi A., Ghanbari M. (2024). Optimizing Concrete Crack Detection: An Attention-Based SWIN U-Net Approach. IEEE Access.

[22] Zou Q., Zhang Z., Li Q., Qi X., Wang Q., Wang S. (2018). Deepcrack: Learning hierarchical convolutional features for crack detection. IEEE Trans. Image Process..

[23] Singha T., Bergemann M., Pham D.S., Krishna A. (2022). SC-CrackSeg: A real-time shared feature pyramid network for crack detection and segmentation. 2022 International Conference on Digital Image Computing: Techniques and Applications (DICTA).

[24] Dosovitskiy A., Beyer L., Kolesnikov A., Weissenborn D., Zhai X., Unterthiner T., Dehghani M., Minderer M., Heigold G., Gelly S. (2020). An image is worth 16x16 words: Transformers for image recognition at scale. arXiv.

[25] Zheng S., Lu J., Zhao H., Zhu X., Luo Z., Wang Y., Fu Y., Feng J., Xiang T., Torr P.H. (2021). Rethinking semantic segmentation from a sequence-to-sequence perspective with transformers. IEEE/CVF Conference on Computer Vision and Pattern Recognition.

[26] Liu Z., Lin Y., Cao Y., Hu H., Wei Y., Zhang Z., Lin S., Guo B. (2021). Swin transformer: Hierarchical vision transformer using shifted windows. Proceedings of the IEEE/CVF International Conference on Computer Vision.

[27] Valanarasu J.M.J., Oza P., Hacihaliloglu I., Patel V.M. (2021). Medical transformer: Gated axial-attention for medical image segmentation. Proceedings of the Medical Image Computing and Computer Assisted Intervention—MICCAI 2021: 24th International Conference, Strasbourg, France, 27 September–1 October 2021.

[28] Inam H., Islam N.U., Akram M.U., Ullah F. (2023). Smart and automated infrastructure management: A deep learning approach for crack detection in bridge images. Sustainability.

[29] Munawar H.S., Ullah F., Shahzad D., Heravi A., Qayyum S., Akram J. (2022). Civil infrastructure damage and corrosion detection: An application of machine learning. Buildings.

[30] Zhang Y., Xu Y., Martinez-Rau L.S., Vu Q.N.P., Oelmann B., Bader S. (2025). On-Device Crack Segmentation for Edge Structural Health Monitoring. arXiv.

[31] Zhang Y., Martinez-Rau L.S., Oelmann B., Bader S. (2024). Enabling autonomous structural inspections with tiny machine learning on UAVs. Proceedings of the 2024 IEEE Sensors Applications Symposium (SAS).

[32] Gu A., Dao T. (2023). Mamba: Linear-time sequence modeling with selective state spaces. arXiv.

[33] Liu J., Yang H., Zhou H.Y., Yu L., Liang Y., Yu Y., Zhang S., Zheng H., Wang S. (2024). Swin-UMamba†: Adapting Mamba-based vision foundation models for medical image segmentation. IEEE Trans. Med. Imaging.

[34] Du W.L., Gu Y., Zhao J., Zhu H., Yao R., Zhou Y. (2024). A mamba-diffusion framework for multimodal remote sensing image semantic segmentation. IEEE Geosci. Remote Sens. Lett..

[35] Zuo X., Sheng Y., Shen J., Shan Y. (2024). Topology-aware mamba for crack segmentation in structures. Autom. Constr..

[36] Chen Z., Shamsabadi E.A., Jiang S., Shen L., Dias-da Costa D. (2024). Vision Mamba-based autonomous crack segmentation on concrete, asphalt, and masonry surfaces. arXiv.

[37] Cai W., Wang X., Xue Y., Ma Y., Wu J., Ge Z., Wang B. (2025). CrackMamba with Normalized Soft-Frangi-Filter Enhancement towards Accurate Crack Segmentation. Proceedings of the 2025 International Conference on Multimedia Retrieval.

[38] Bansal S., Madisetty S., Rehman M.Z.U., Raghaw C.S., Duggal G., Kumar N. (2024). A comprehensive survey of mamba architectures for medical image analysis: Classification, segmentation, restoration and beyond. arXiv.

[39] Rahman M.M., Tutul A.A., Nath A., Laishram L., Jung S.K., Hammond T. (2024). Mamba in vision: A comprehensive survey of techniques and applications. arXiv.

[40] Zhang Y., Liu H., Hu Q. (2021). Transfuse: Fusing transformers and cnns for medical image segmentation. Proceedings of the International Conference on Medical Image Computing and Computer-Assisted Intervention.

